# Samp1 Mislocalization in Emery-Dreifuss Muscular Dystrophy

**DOI:** 10.3390/cells7100170

**Published:** 2018-10-15

**Authors:** Elisabetta Mattioli, Marta Columbaro, Mohammed Hakim Jafferali, Elisa Schena, Einar Hallberg, Giovanna Lattanzi

**Affiliations:** 1 CNR Institute of Molecular Genetics, Unit of Bologna, 40136 Bologna, Italy; emattiol@area.bo.cnr.it (E.M.); elisaschena83@gmail.com (E.S.); 2 IRCCS Istituto Ortopedico Rizzoli, 40136 Bologna, Italy; 3 Laboratory of Musculoskeletal Cell Biology, IRCCS Istituto Ortopedico Rizzoli, 40136 Bologna, Italy; marta.columbaro@ior.it; 4 KTH, Science for Life Laboratory, SE 171 65 Solna, Sweden; hakim.mohmd@scilifelab.se; 5 Endocrinology Unit, Department of Medical & Surgical Sciences, Alma Mater Studiorum University of Bologna, S Orsola-Malpighi Hospital, 40138 Bologna, Italy; 6 Department of Biochemistry and Biophysics (DBB), Stockholm University, SE-106 91 Stockholm, Sweden; einar.hallberg@neurochem.su.se

**Keywords:** Emery-Dreifuss Muscular Dystrophy type 2 (EDMD2), Samp1 (NET5), prelamin A, LINC complex, myonuclear positioning

## Abstract

*LMNA* linked-Emery-Dreifuss muscular dystrophy (EDMD2) is a rare disease characterized by muscle weakness, muscle wasting, and cardiomyopathy with conduction defects. The mutated protein lamin A/C binds several nuclear envelope components including the Linker of Nucleoskeleton and Cytoskeleton (LINC) complex and the inner nuclear membrane protein Samp1 (Spindle Associated Membrane Protein 1). Considering that Samp1 is upregulated during muscle cell differentiation and it is involved in nuclear movement, we hypothesized that it could be part of the protein platform formed by LINC proteins and prelamin A at the myotube nuclear envelope and, as previously demonstrated for those proteins, could be affected in EDMD2. Our results show that Samp1 is uniformly distributed at the nuclear periphery of normal human myotubes and committed myoblasts, but its anchorage at the nuclear poles is related to the presence of farnesylated prelamin A and it is disrupted by the loss of prelamin A farnesylation. Moreover, Samp1 is absent from the nuclear poles in EDMD2 myotubes, which shows that *LMNA* mutations associated with muscular dystrophy, due to reduced prelamin A levels in muscle cell nuclei, impair Samp1 anchorage. Conversely, SUN1 pathogenetic mutations do not alter Samp1 localization in myotubes, which suggests that Samp1 lies upstream of SUN1 in nuclear envelope protein complexes. The hypothesis that Samp1 is part of the protein platform that regulates microtubule nucleation from the myotube nuclear envelope in concert with pericentrin and LINC components warrants future investigation. As a whole, our data identify Samp1 as a new contributor to EDMD2 pathogenesis and our data are relevant to the understanding of nuclear clustering occurring in laminopathic muscle.

## 1. Introduction

The nuclear envelope is linked to chromatin on the nucleoplasmic side and cytoskeleton on the outer side [1]. The connection between compartments is provided by the nuclear lamina and the LINC complexes formed by interactions between KASH domains of proteins traversing the outer nuclear membrane (nesprins) and the SUN domains of SUN proteins, which are located in the perinuclear space [2,3]. The LINC complex is conserved from yeast to mammals and has been found to have essential roles in many cell functions including cell polarization, nuclear migration and positioning [4].

The latter process plays a key role in differentiating neuronal [5] and muscle cells [6] and requires centrosome anchorage to the nuclear envelope and microtubules [7,8,9]. Consistently, loss of centrosome function and altered nuclear movement have been linked both in experimental models [10] and in human muscular diseases [11,12,13]. It has been demonstrated that nesprin-2G, SUN2, and lamin A/C are indispensable for centrosome orientation and nuclear movement in muscle cells [14] and LINC complex proteins defects cause altered myonuclear positioning in muscular dystrophies [13,15,16,17]. These data have been confirmed in experimental models [15,17] and the involvement of other KASH domain proteins has been demonstrated [11,18,19,20]. Moreover, we have identified pericentrin as the first centrosomal protein affected in muscular dystrophy and, in particular, in cells bearing SUN1 mutations [17].

Anchoring centrosomes close to the nucleus was also shown to depend on the inner nuclear membrane protein Samp1 [21]. In fact, Samp1 directs localization of gamma-tubulin, which is a major component of centrosomal complexes [22]. Furthermore, Samp1 interacts with LINC proteins SUN2, SUN1, emerin, and A-type lamins [23,24,25] and interferes with nuclear movement [26]. Samp1 is required for muscle cell differentiation, which was demonstrated in mouse myoblasts and human induced pluripotent stem cells [27,28]. In this context, we consider particularly important the nuclear poles and the nuclear envelope interplay with centrosomal proteins. We previously observed that the opposite poles of myonuclei in most cases located along the major longitudinal axis of myoblasts and myotubes are characterized by a specific composition of the nuclear envelope with enrichment of SUN2 and farnesylated prelamin A [15]. Nuclear movement is oriented and the position of the nuclear poles is in relation with that of the centrosome. The nuclear poles’ position is usually behind it during nuclear movement. Microtubule nucleation occurs from the nuclear envelope in myotubes. Thus, the loss of the nuclear envelope integrity affects microtubule organization and nuclear positioning [17].

We had previously demonstrated that farnesylated prelamin A is necessary to recruit SUN1 to the nuclear envelope of differentiating muscle cells and it is required for the accumulation of SUN2 at the nuclear poles [15]. In that study, we compared normal human myoblasts with cells from type 2 Emery-Dreifuss muscular dystrophy (EDMD2), which is a disorder caused by *LMNA* mutations and characterized by muscle weakness, muscle wasting, and cardiomyopathy [15,29]. We found that reduced prelamin A levels in EDMD2 muscle impair SUN1 recruitment to the nuclear envelope and accumulation of SUN2 at the nuclear poles and cause myonuclear clustering [15]. Clustering of myonuclei was also observed in muscle cells bearing SUN1 mutations, which supports the view that lamins and LINC complex proteins are required for proper myonuclear positioning [17]. In fact, movement and anchorage of myonuclei during myogenesis or muscle injury repair are regulated through complex mechanisms, but are not fully elucidated [6,15,17,19].

In this study, we hypothesized that Samp1 could be part of the protein platform formed by LINC proteins and prelamin A at the myotube nuclear envelope and, as previously demonstrated for those proteins, could be affected in EDMD2. Our results show that Samp1 is recruited to the nuclear envelope of human myotubes and myoblasts committed to differentiation and persists in mature human muscle, which supports previous data obtained in murine cells [27]. Although we observe a uniform distribution of Samp1 in the nuclear envelope, loss of prelamin A farnesylation [15] causes Samp1 mislocalization from the nuclear poles. Moreover, in EDMD2 myotubes, Samp1 is overall preserved at the nuclear envelope, which is reported in Reference [30], but it is missing from the nuclear poles. Thus, a protein platform including farnesylated prelamin A, SUN proteins, and Samp1 is located at the nuclear poles of myonuclei and it is disrupted in EDMD2. Samp1 loss from the nuclear poles might contribute to an altered interplay of the nucleus with cytoskeleton or centrosomal constituents in EDMD2.

## 2. Materials and Methods

### 2.1. Cell Cultures and Treatments

Control and EDMD2 myoblast cultures were established, as previously described [15], from muscle biopsies of consenting patients according to local and EU ethical rules. Cells were cultured in D-MEM plus 20% fetal calf serum and antibiotics. To obtain myotubes, confluent myoblast cultures at 100% confluence were kept in culture medium for 7 to 10 days. In the EDMD2 myoblasts used in this study, the R190Q/R249Q *LMNA* mutations had been determined in a single allele [31]. Moreover, we used myoblasts from an EDMD2 patient harboring the H506P *LMNA* mutation [32] and a muscular dystrophy linked to the compound heterozygous P68D/G338S *SUN1* mutation [17]. Accumulation of non-farnesylated prelamin A was obtained after 18 h of treatment of differentiating myoblasts with 20 µM Mevinolin (Sigma).

### 2.2. Muscle Biopsies

Skeletal muscle biopsies from healthy subjects and an EDMD2 patient bearing the H506P *LMNA* mutation were frozen in melting isopentane and stored in liquid nitrogen. Cryo-sections were fixed in 2% paraformaldehyde, permeabilized with 0.05% Triton X-100 in PBS, and subjected to immunofluorescence staining.

### 2.3. Immunofluorescence Analysis

Cells fixed in 4% paraformaldehyde were treated with 0.15% Triton X-100 and stained according to previously published protocols [15]. The following primary antibodies were used: anti-Samp1 (from Hallberg laboratory); anti-lamin B and anti-pericentrin from Abcam (Cambridge, UK); anti-desmin and anti-emerin from Monosan (Uden, The Netherlands); anti-SUN1 and anti-SUN2 from Sigma (St. Louis, MO, USA); anti-farnesylated prelamin A (1188-2) from Diatheva (Pesaro, Italy), anti-caveolin 3 from BD Transduction (San Francisco, CA, USA); anti-prelamin A (Sc-6214) form Santa Cruz Biotechnology (Dallas, TX, USA), and anti-laminin alpha2 from Chemicon (Massachusetts, MA, USA). Image acquisition was performed by using a Nikon Eclipse Ni epifluorescence microscope equipped with a digital CCD camera and NIS-Elements 4.3 AR software. Photoshop CS was used for image processing. Mean fluorescence intensity was measured by using NIS-Elements 4.3 AR.

### 2.4. Proximity Ligation Assay (PLA)

In-situ PLA was performed by using the Duolink Fluorescence kit (Duolink^®^ In Situ Red Starter Kit Goat/Rabbit) from Sigma (St. Louis, MO, USA), according to previously described protocols [33]. For PLA, anti-Samp1 and anti-prelamin A antibodies were applied to human myotube cultures. Image acquisition was performed by using the Nikon Eclipse Ni fluorescence microscope equipped with a digital CCD camera and NIS Elements AR 4.3 software. PLA signals were counted by using the Duolink ImageTool software from Sigma (St. Louis, MO, USA). Duolink ImageTool is a dedicated and user-friendly software, specifically designed for objective quantification/counting of PLA signals in images generated from fluorescence microscopy. The nuclei are automatically detected and cytoplasm size estimated, enabling single cell statistical analysis of expression levels in tissue or cell populations.

### 2.5. Statistical Analysis

All data except PLA were obtained in three diverse control myoblast cultures and in both EDMD2 myoblast cultures bearing the *LMNA* mutations described above. At least 100 nuclei were counted or measured for fluorescence intensity unless differently stated in figure legends. Data obtained from three different experiments were analyzed by using the Mann-Whitney non-parametric test and reported in graphs as a mean of three independent experiments ± standard error. A *p* value < 0.05 was considered as statistically significant.

## 3. Results

**Figure 1:**
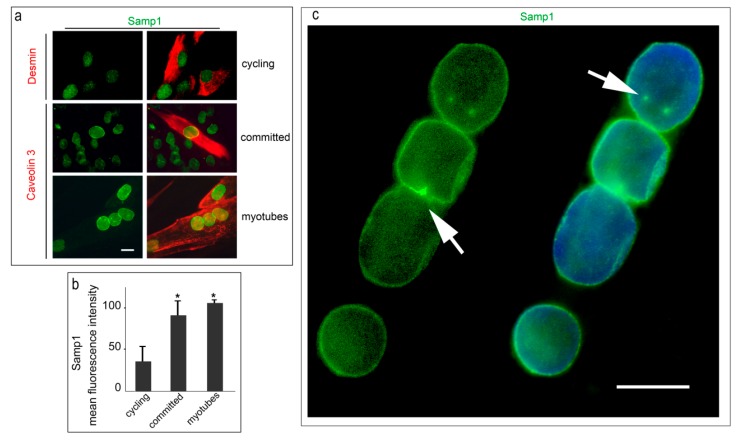
Samp1 is recruited to the nuclear periphery in differentiating human control myoblasts. (**a**) Samp1 (green) and desmin (red) were co-stained in cycling human myoblasts (cycling). Samp1 and caveolin 3 (red) were co-stained in committed myoblasts (committed) and myotubes (myotubes). Nuclear envelope localization of Samp1 is observed in all caveolin 3-positive cells. (**b**) Quantitative analysis of mean fluorescence intensity is reported in the graphs. (**c**) A myotube labeled with Samp1 antibody and counterstained with DAPI shows a few sites of accumulation of Samp1 in areas possibly corresponding to centrosome remnants (arrows). Statistically significant differences by the Mann Whitney test are indicated by asterisks (*). Scale bars, 10 µm.

The increase of Samp-1 levels and recruitment to the nuclear rim was observed in committed human myoblasts (caveolin 3-positive mononucleated cells) and myotubes (caveolin 3-positive multi-nucleated cells) (Figure 1a,b). The nuclear envelope is a microtubule organizing center in myotubes [17,33]. Centrosome proteins are re-localized during myogenesis and some of them including pericentrin move to the nuclear periphery in myotubes and are required for microtubule nucleation [17]. In this case, we looked carefully at Samp1 localization in myotubes and found that the protein was accumulated at discrete points (Figure 1c), which may possibly correspond to centrosome remnants [33].

**Figure 2:**
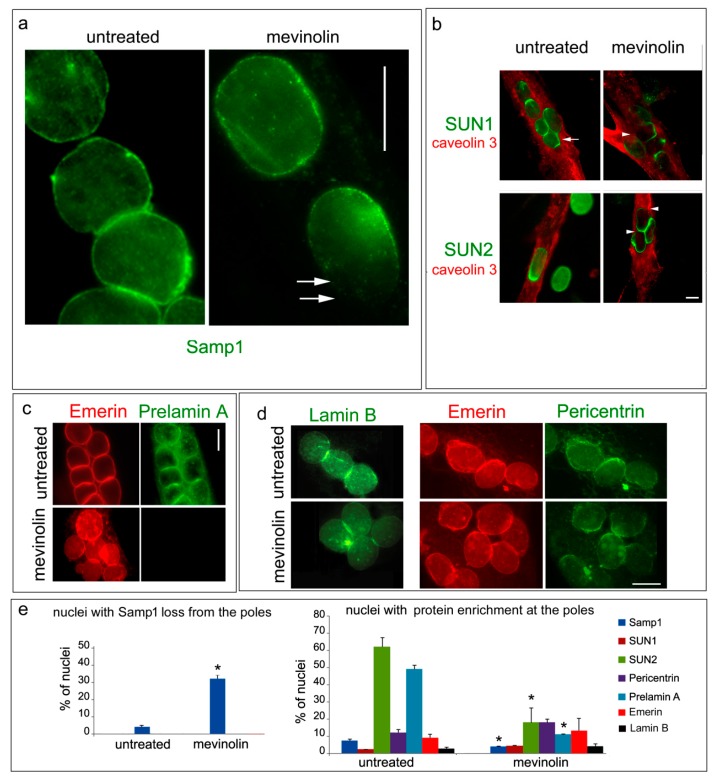
Loss of Samp1 from the nuclear poles in the absence of farnesylated prelamin A. Human control myotubes left untreated (untreated) or treated with mevinolin (mevinolin) are shown. (**a**) Samp1 staining in myotubes. Arrows show a nuclear pole devoid of Samp1 in a myotube subjected to mevinolin. (**b**) Co-staining of SUN1 or SUN2 with caveolin 3. SUN1 and SUN2 loss from the nuclear poles is observed in mevinolin-treated myotubes. The arrow points to a nuclear pole connected to a nucleus of a fusing myoblast. Arrowheads show protein loss from a nuclear pole. (**c**) Human myotubes co-stained for emerin and farnesylated prelamin A (Diatheva 1188-2 antibody). The antibody used to detect prelamin A is specific for farnesylated prelamin A, which is not detectable in mevinolin treated cells. (**d**) Staining of lamin B (**left** panel) and co-staining of emerin and pericentrin (**right** panel) in myotubes. (**e**) Quantitative analysis of the percentage of nuclei showing loss of Samp1 staining at the nuclear pole(s) (**left** panel) and protein accumulation at the nuclear poles (**right** panel) in untreated or mevinolin-treated cells. Mean values of three counts are reported in the graphs. Statistical significance of differences (*p* < 0.05) is indicated by an asterisk (*). Scale bars, 10 µm.

We previously showed that inhibition of prelamin A farnesylation impairs SUN1 recruitment to the nuclear envelope and accumulation of SUN2 at the nuclear poles of differentiated muscle cells [15]. Although Samp1 was evenly distributed along the nuclear envelope of human myotubes as shown in Figure 1c and Figure 2a, we decided to investigate whether loss of farnesylated prelamin A could affect Samp1 anchorage. In myoblasts and myotubes that lost farnesylated prelamin A due to mevinolin treatment [15], Samp1 was mislocalized and its levels were reduced at the nuclear poles (Figure 2a). These results suggested that lamin A anchorage was required for proper localization of Samp1 in muscle cells and anchorage at the nuclear poles was dependent on farnesylated prelamin A, as seen in SUN1 and SUN2 [15]. In fact, as previously shown, SUN2 was not accumulated at the nuclear pole of mevinolin-treated nuclei and SUN1 was mis-localized in a percentage of nuclei (Figure 2b). However, we did not observe loss of emerin staining at the nuclear poles of myotubes in the absence of prelamin A farnesylation even though dysmorphic nuclei with uneven distribution of emerin were observed (Figure 2c). Additionally, lamin B fluorescence intensity and protein localization in myonuclei were not affected by mevinolin (Figure 2d).

Samp1 has been recently shown to interact with gamma-tubulin, which is a major part of centrosomes [22]. In this case, we wanted to check the fate of pericentrin, which is a major binding partner of gamma-tubulin that is associated with the centrosome in proliferating myoblasts and moves to the nuclear periphery in myotubes [17,33]. Figure 2d shows localization of pericentrin at the nuclear periphery and accumulation at the nuclear poles of myonuclei [17]. Pericentrin localization is preserved in mevinolin-treated cells even though, in drug-treated myotubes, a percentage of pericentrin negative nuclear poles is observed (Figure 2d). The evaluation of these results (Figure 2d) suggests that prelamin A affects Samp1 as well as LINC protein localization in a significant percentage of myotube nuclei.

**Figure 3:**
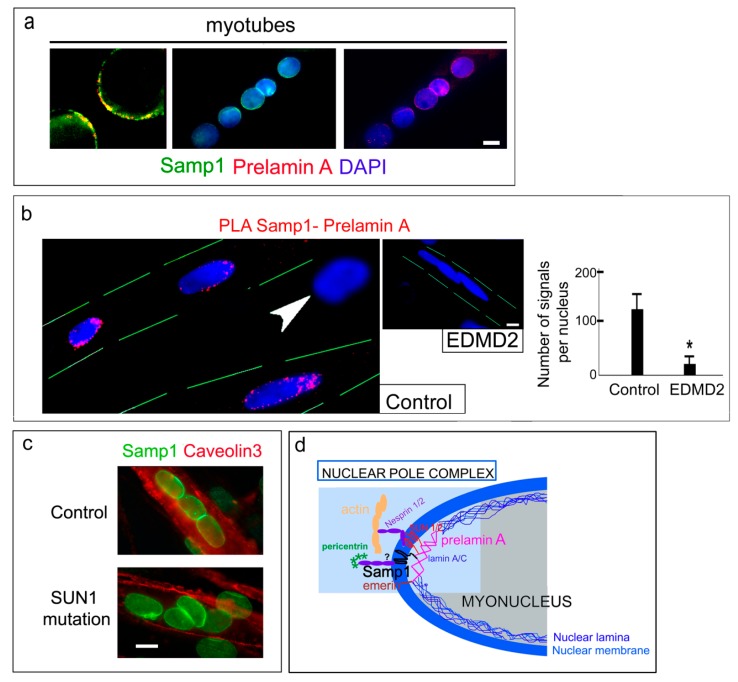
Samp1 co-localizes with farnesylated prelamin A at the nuclear poles of human myotubes. (**a**) Co-staining of Samp1 and prelamin A in human control myotubes (myotubes). Left panel, high magnification of a double stained nuclear pole showing protein colocalization. Single fluorescence of Samp1 and prelamin A are shown along with DAPI staining of nuclei. (**b**) PLA of prelamin A and Samp1 (PLA Prelamin A-Samp1, red dots) in control myotubes. The arrowhead indicates a mononucleated myoblast that is negative for PLA. An EDMD2 myotube negative for PLA is shown in the inset. Graphs show quantitative analysis of PLA signals reporting the average number of spots in nuclei (80 nuclei per sample were counted). (**c**) Immunostaining of Samp1 and Caveolin 3 in control myotubes (control) and myotubes from a muscular dystrophy caused by *SUN1* mutation (SUN1 mutation, see methods for details) [17]. (**d**) Schematic representation of the proposed protein platform (myo-Nuclear Pole Complex) at the nuclear envelope of differentiated human myoblasts. Prelamin A interacts with Samp1, SUN1/2, and emerin. SUN1/2 link the complex to nesprins and the actin cytoskeleton. The hypothesis (indicated by “?”) that Samp1 binds nesprin 2G and pericentrin warrants future investigation. Statistically significant differences by the Mann Whitney test are indicated by asterisks (*). Nuclei are counterstained with DAPI (blue). Scale of bars, 10 µm.

Thus, Samp1 localization at the nuclear poles of myotubes appeared to be dependent on prelamin A interaction. In support of this hypothesis, we observed co-localization (Figure 3a) and a proximity ligation assay (PLA) positivity of prelamin A and Samp1 in human normal myotubes mostly at the nuclear poles (Figure 3b). These findings suggested that the “pole structure” previously described in human muscle cells included not only farnesylated prelamin A, SUN1, and SUN2 but also Samp1. However, while pathogenetic *LMNA* mutations disrupted prelamin A-Samp1 interaction as shown by loss of PLA signals in EDMD2 myotubes (Figure 3b), pathogenetic SUN1 mutations did not affect Samp1 localization in myotubes (Figure 3c).

Thus, mutations in *LMNA* alter both Samp1 (this study) and SUN2 localization at the poles of myonuclei [15], while SUN1 mutations affect SUN2 recruitment [17] and do not impair Samp1 localization. Based on these considerations, we propose a model (depicted in Figure 3d) of protein-protein interaction at the nuclear poles of human myonuclei. In this model, prelamin A anchors Samp1 and SUN1 at the nuclear poles. Loss of prelamin A occurs in EDMD2 and impairs Samp1 anchorage. 

**Figure 4:**
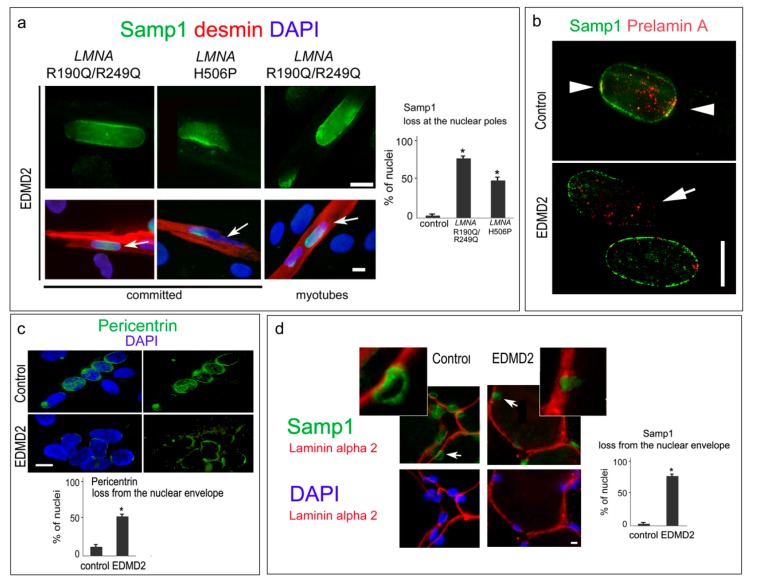
Mis-localization of Samp1 in the EDMD2 nuclei. (**a**) Samp1 staining in EDMD2 myoblasts committed to differentiation (committed) and myotubes (myotubes). Myotubes are double stained for desmin (red). Upper panel, high magnification of nuclei indicated by arrows. The percentage of nuclei showing Samp1 loss at the nuclear poles (at least one pole) is reported in the graph. (**b**) Double staining of prelamin A and Samp1 in differentiated control and EDMD2 myoblasts. Arrowheads indicate co-localization, the arrow indicates a nuclear pole devoid of Samp1 and prelamin A fluorescence. (**c**) Pericentrin staining in the control and the EDMD2 myotubes. Percentage of nuclei showing pericentrin loss at the nuclear periphery (negative nuclei) is reported in the graph. (**d**) Samp1 and laminin alpha 2 were co-stained in muscle tissue from control (control) and EDMD2 (EDMD2). Nuclei inside the basal lamina outlined by laminin alpha 2 are myonuclei. Percentage of nuclei showing Samp1 loss from the nuclear envelope is reported in the graph. Magnification of selected nuclei (arrows) is shown in the insets. Nuclei in (**a**,**c**,**d**) are counterstained with DAPI. Statistically significant differences by the Mann Whitney test are indicated by asterisks (*). Scale of bars, 10 µm.

Based on the above reported results, we evaluated Samp1 localization in EDMD2 cells and muscle tissue. The focal loss of Samp1 from the nuclear envelope, mostly from the nuclear poles, was observed in EDMD2 myoblasts committed to differentiation and in myotubes (Figure 4a). Overall reduction of prelamin A fluorescence intensity was observed in 40% to 50% of EDMD2 myoblasts, as reported in Reference [15], and prelamin A was undetectable in the nuclear poles devoid of Samp1 staining (Figure 4b).

We also observed significant reduction of pericentrin fluorescence intensity at the nuclear periphery of EDMD2 myotubes (Figure 4c), which suggests that *LMNA* mutations impair pericentrin recruitment during myotube formation as previously observed in SUN1 and nesprin 1 mutant cells [13,17]. This finding is relevant in view of the reported interplay of Samp1 with centrosomal constituents. In EDMD2 mature muscle, we observed mis-localization of Samp1 from the nuclear envelope of myonuclei. In fact, double immunolabeling of Samp1 and laminin alpha 2, which lines the basal lamina of myofibers, demonstrated that nuclei inside control muscle fibers were Samp1 positive and the protein was located at the nuclear periphery, which was previously reported in Reference [30], while interstitial nuclei presented a faint staining (Figure 4d). In EDMD2 myonuclei bearing the H506P *LMNA* mutation [32], Samp1 was mis-localized in the vast majority (80%) of examined myofibers (Figure 4d) possibly due to a loss of interaction with farnesylated prelamin A, which is dramatically reduced in EDMD2 muscle fibers [15].

## 4. Discussion

Muscular dystrophies are caused by mutations in a number of apparently unrelated genes [34]. However, they cause, in most cases, muscle weakness and wasting and a typical disproportion of myofibers with fibrosis and fat substitution. These considerations suggest that common pathogenetic mechanisms might be involved in muscular dystrophies. Altered anchorage of myonuclei and myonuclear clustering have been linked to the pathogenesis of muscle disorders associated or not with mutations in nuclear envelope proteins [13,15,17,35]. Nuclear positioning is particularly important in muscle cells during differentiation since uneven nuclear spacing causes the formation of myonuclear domains of different sizes, which affects muscle function [6,36]. The list of nuclear envelope and LINC proteins involved in nuclear positioning in muscle includes prelamin A [15], emerin [37,38], SUN1, SUN2 [15,18], and nesprins [39], which are major players on the cytoplasmic side [13,39]. Our results add Samp1 as a new component of a complex required at the nuclear poles of differentiating myoblasts and is most likely aimed at centrosome interplay during nuclear movement [21].

During myogenic differentiation, prelamin A is increased at the nuclear envelope [40] in its farnesylated form [15] and it is accumulated at the nuclear poles. Accumulated prelamin A recruits SUN1 and directs accumulation of SUN2 at nuclear poles [15]. This is an area where key centrosomal proteins are found during myogenesis [13,17,33]. Since loss of Samp1 from the nuclear poles is elicited by the impairment of prelamin A farnesylation in normal human myotubes, we suggest that farnesylated prelamin A is also required to anchor Samp1 in the nuclear poles of differentiated muscle cells. In support of this hypothesis, enrichment of prelamin A-Samp1 PLA signals is measured at the nuclear poles of myonuclei. SUN1, SUN2, and prelamin A polarization are required for proper positioning of myonuclei. Thus, loss of polarized proteins has been linked to the presence of clustered nuclei [15,17]. These nuclei may often appear as a single elongated nucleus in the skeletal and cardiac muscle of patients affected by EDMD2 or dilated cardiomyopathy (DCM-CD) [16].

Importantly, although in the majority of myonuclei Samp1 is evenly distributed in the nuclear envelope, mutations in *LMNA* disrupt Samp1 localization specifically at the nuclear poles. Our results are in agreement with published data showing reduced Samp1 levels at the nuclear envelope of cells depleted of lamin A/C [26] and reinforce the concept that Samp1 interplay with A type lamins is functionally relevant for myogenesis. However, in agreement with the observation that Samp1 binds mature lamin A [26], the nuclear envelope protein is retained in the nuclear rim out of the nuclear poles after inhibition of prelamin A farnesylation, which is most likely through mature lamin A binding. In EDMD2 mature muscle, Samp1 is almost completely mis-localized from the nuclear envelope with a low percentage of negative nuclei. A previous study [30] showed that Samp1 is correctly localized in myofibers from EDMD2 patients. As for other nuclear envelope proteins analyzed by the same authors [30], the different outcome of our Samp1 study may be related to the different *LMNA* mutation. In particular, the heterozygous compound R190Q/R249Q *LMNA* mutation associated in the patient with severe cardiomyopathy that leads to heart transplantation and severe skeletal muscle wasting [31], might disrupt Samp1 anchorage by affecting two lamin A/C domains. However, the H506P *LMNA* mutation also caused Samp1 mis-localization from the nuclear poles, which suggests that reduction of lamin A and/or prelamin A levels plays a major role in Samp1 loss in laminopathic muscle. In this study, we found that lamin B and emerin were not reduced at the nuclear poles of EDMD2 myotubes even though they showed an unordered distribution at the nuclear envelope. To fully elucidate the relevance of Samp1 in EDMD2 pathogenesis, it will be relevant to test the interplay between Samp1 and mutated lamin A/C as well as Samp1 interplay with emerin, lamin B, and LAP2 alpha in EDMD2 muscle [41,42,43].

The data reported in this paper and previous findings allow us to state that, in the EDMD2 myonuclear envelope, (1) prelamin A levels and SUN1 interaction are reduced, (2) SUN2 and prelamin A are not enriched at the nuclear poles, and (3) Samp1 is absent from most of the nuclear poles.

These data and the reported clustering of myonuclei caused by pathogenetic *LMNA* mutations strengthen the hypothesis that the nuclear poles of differentiating muscle cells hold a protein complex (including the LINC complex proteins) aimed at an interaction with centrosomal proteins for proper nuclear movement and positioning. Several papers show altered myonuclear positioning in cells devoid of *LMNA* [11], SUN1/SUN2 [18], or nesprin [13,39]. We have observed a striking clustering of myonuclei in myotubes bearing heterozygote compound mutations in SUN1 [17]. Those cells have been obtained from a patient affected by an EDMD-like muscular dystrophy with cardiac involvement. Importantly, in SUN1-mutated myotubes showing myonuclear clustering, the centrosomal protein pericentrin fails to localize at the nuclear periphery. In this paper, we report that pericentrin levels are also reduced in EDMD2 myotubes. Given its involvement in centrosome complexes and nuclear movement, Samp1 is an obvious candidate for pericentrin functional interaction.

A number of published papers show that mutations in lamins disrupt localization of lamin binding partners at limited areas of the nuclear envelope. For instance, lamin B loss from the nuclear blebs formed in laminopathic cells has been reported in familial partial lipodystrophy [44], EDMD2 [45], and cells from atypical-progeria [46]. This phenomenon has been interpreted as due to the existence of separate, but interacting, lamin microdomains with diverse functions in chromatin organization and transcription [47]. In this paper, we propose the existence of a new lamin microdomain, the myo-nuclear pole complex, as a specialized protein platform in muscle nuclei and affected in muscle diseases of the nuclear envelope. Defects in Samp1 and its binding partners could impair nuclear movement occurring during myoblast differentiation [15] or reduce the myoblast ability to adapt to the forming extracellular matrix [3]. This is a topic that deserves further studies.
